# Supplementary material for: Ikaros family zinc finger 1 regulates dendritic cell development and function in humans
Source: Nat Commun. 2018 Mar 27;9:1239. doi: 10.1038/s41467-018-02977-8 (PMC5869589; doi:10.1038/s41467-018-02977-8)
Supplement: Supplementary file 1 — Supplementary Information [file 41467_2018_2977_MOESM1_ESM.pdf]

Ikaros family zinc finger 1 regulates dendritic cell development and function in humans: supplementary information

Cytlak et al

Supplementary Figure 1

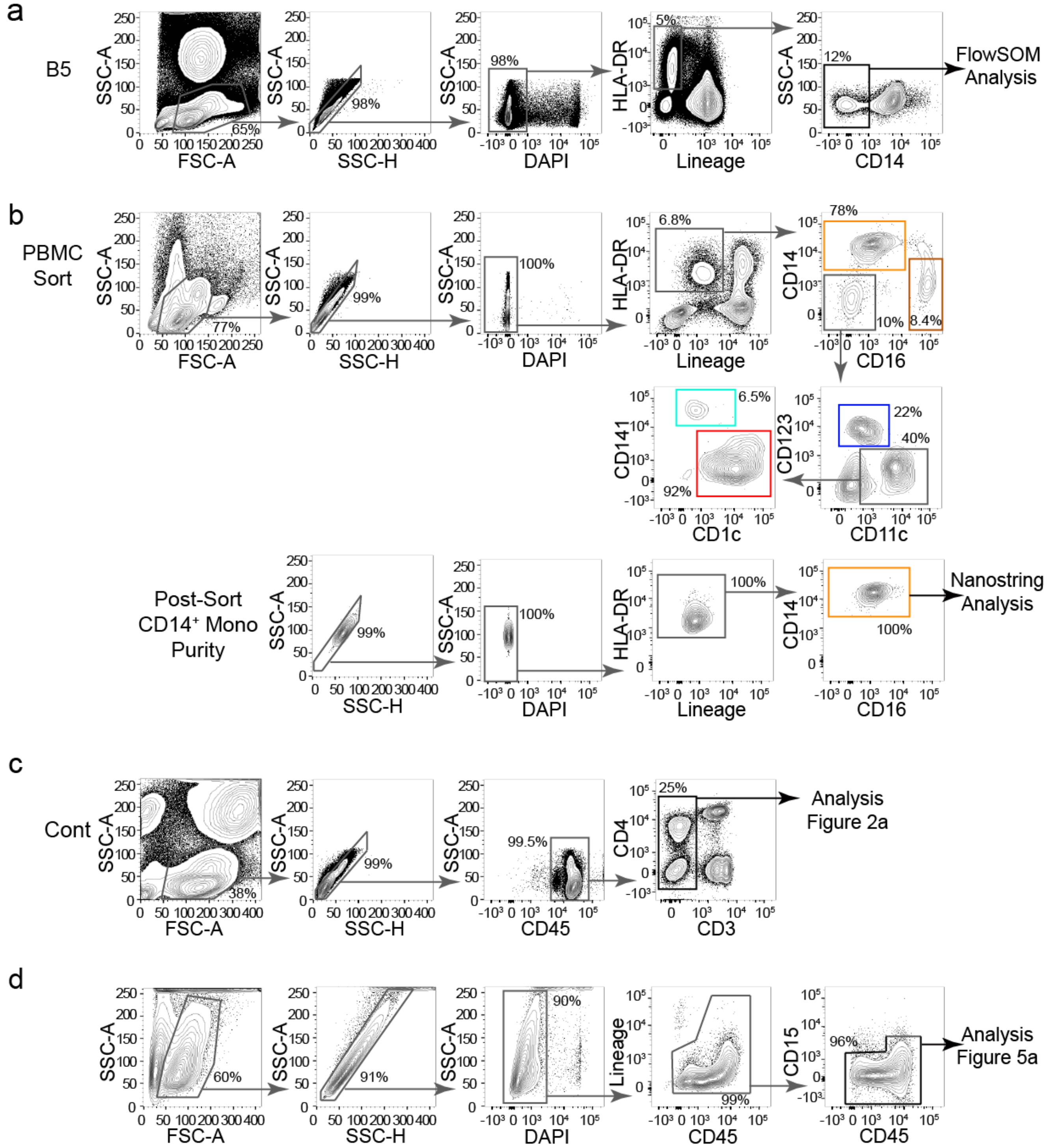

Supplementary figure 1: Flow cytometric gating strategies

In all flow cytometric analyses, PBMC were identified by SSC and FSC properties, doublets excluded and live (DAPI/ZOMBIE negative) cells gated for subsequent analysis.

(a) Identification of cells used in FlowSOM analysis gated sequentially as; cells, singlets, live cells, Lineage$^-$ (CD3,7,16,19,20,34) HLA-DR$^+$ and CD14$^-$. (b) FACS sorting strategy for NanoString *IKZF1* gene expression analysis: within the Lineage$^-$ (CD3,19,20,56) HLA-DR$^+$ gate were found CD14$^+$ monocytes (orange), CD16$^+$ monocytes (brown), CD123$^+$ pDC (blue), CD141$^+$ cDC1 (turquoise), CD1c$^+$ cDC2 (red). Post-sort purity of CD14$^+$ monocytes was >98%. (c) Upstream gating identifying CD45$^+$CD3$^-$ cells for Trucount analysis (Figure 2a). (d) Upstream gating for analysis of *in vitro* CD34$^+$ progenitor culture output (Figure 5a) identifying CD45$^+$CD15$^-$ cells for subsequent analysis.

Supplementary figure 2

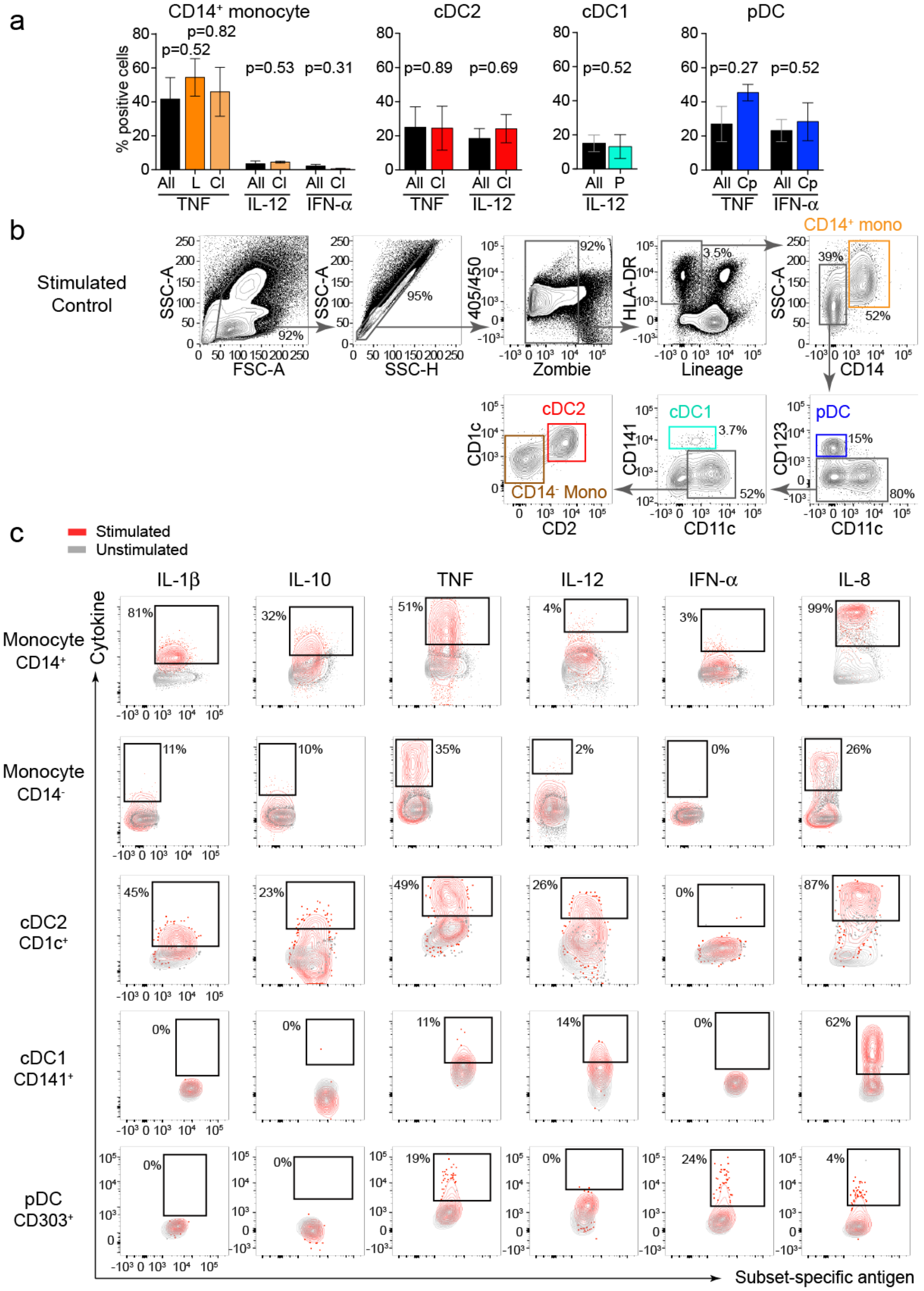

Supplementary figure 2: Flow cytometric gating strategy for DC functional assay
(a) Percentage of cells staining positive for the specified cytokine with cocktail (All) or individual TLR agonist (L, LPS; Cl, CL075; P, PolyI:C; Cp, CpG) in CD14$^+$ monocytes (orange), cDC2 (red), cDC1 (turquoise) and pDC (blue). p values generated by paired, two-tailed t test. (b) Gating strategy to identify cell subsets for quantification of cytokine production following overnight culture with TLR agonists. The lineage$^-$(CD3,16,19,20) HLA-DR$^+$ population was identified from within the live, singlet cells. This fraction contained CD14$^+$ monocytes (orange), CD14$^-$ monocytes (brown), CD123$^+$ pDC (blue), CD141$^+$ cDC1 (turquoise) and CD2$^+$CD1c$^+$ cDC2 (red). (c) Cytokine production was quantified as the percentage of positive cells/parent population. Cytokines are displayed by columns (y-axis) and cell subsets are displayed by rows with the subset specific antigen denoted in the row title displayed on the x-axis (CD14 for monocytes, CD1c for cDC2, CD141 for cDC1 and CD303 for pDC).

Supplementary table 1: Patient details

Family	Patient	Mutation	CD14+ Mono	CD16+ Mono	pDC	cDC1	cDC2	Symptomatic	ivIg	B cell (previous)	B cell (current)
B	B5	R162Q	258.60	**0.08**	**2.40**	**3.20**	15.11	Infections	Yes	**50**	**48**
	B6	R162Q	376.87	**7.05**	**3.70**	**3.00**	15.90	Infections	Yes	**20**	**11**
C	C1	H167R	361.89	**2.95**	**1.50**	**2.08**	12.85	Infections	Yes	**26**	**12**
	C2	H167R	260.94	**3.38**	**2.35**	**1.97**	11.33	Infections	Yes	**5**	**2**
	C3	H167R	203.26	**0.90**	**3.22**	**3.84**	17.21	Infections	Yes	**18**	**9**
	Sib of C2 and C3	WT	466.00	15.00	9.02	0.40	11.80	-	-		138
F	F1	del 7p12.3-p12.1	382.71	**14.32**	**4.74**	**1.72**	19.69	Infections		**11**	**42**
	F3	del 7p12.3-p12.1	251.70	**5.63**	**2.23**	**1.10**	9.15	Infections	Yes	**18**	**5**
	F4	del 7p12.3-p12.1	409.66	**9.30**	**2.88**	**0.72**	14.36	Infections	Yes	**8**	**2**
	F5	del 7p12.3-p12.1	434.90	**11.29**	**4.91**	**0.94**	10.02	Infections	No	**12**	**1**
	F6	del 7p12.3-p12.1	354.41	**4.38**	**4.70**	**0.50**	9.13	Infections	No	138	**75**
	F7	del 7p12.3-p12.1	332.23	**9.15**	**7.75**	**1.39**	10.99	No	No	**9**	**5**
	F8	del 7p12.3-p12.1	238.86	**8.01**	**4.73**	**1.31**	12.32	No	No	**89**	**100**
	F9	del 7p12.3-p12.1	355.12	**9.60**	**1.11**	**0.57**	6.75	No	No	354	310
	F10	del 7p12.3-p12.1	410.72	**7.68**	**2.07**	**1.45**	11.30	No	No	187	144
	F11	del 7p12.3-p12.1	305.31	**12.71**	**7.38**	**0.91**	19.46	No	No	544	490
	F12	del 7p12.3-p12.1	426.95	**5.95**	**6.18**	**1.57**	19.03	B-ALL and infections	No	452	431
	F13	del 7p12.3-p12.1	480.82	**3.82**	**2.19**	**1.16**	9.97	No	No	291	170
	WT Sib of F9 'F4a'	WT	241.78	18.72	6.03	0.51	14.78	-	-		457
	WT Sib of F9 'F4b'	WT	277.72	17.98	11.60	0.68	12.23	-	-		491
G	G1	S46Afs*14	260.94	**11.58**	**2.36**	**3.70**	11.33	No	No	141	101
	G2	S46Afs*14	203.26	**19.16**	**3.40**	**1.98**	17.21	Infections/autoimmunity	No	**17**	**4**
	G3	S46Afs*14	361.89	**20.99**	**1.84**	**1.70**	12.85	Infections/autoimmunity	No	**122**	**85**
	Sib of G2 and G3	WT	222.00	23.93	8.00	1.10	17.30	-	-		403

Supplementary table 1: IKZF1 haploinsufficiency patient details

Details of affected and unaffected members of Families B, C, F[25] and G[27] recruited to the study describing: *IKZF1* mutation, monocyte and DC enumeration, presence of symptoms, use of intravenous immunoglobulin infusion (ivIg), $CD19^+$ B cell counts (previous and current), mono, monocytes; pDC, plasmacytoid DCs; cDC1/2, conventional DC1/2. Absolute counts were derived from gating described in Figure 2. Absolute counts expressed as cells/μL. Data pertinent to time of DC/monocyte analysis except previous B cell counts, as documented in Ref [25].

Supplementary table 2: Antibodies used for Flow Cytometry

Antigen	Fluorochrome	Clone	Dilution	Manufacturer
**BTLA**	**PECF594**	J168-540	5	BD
**CD11c**	BV421/**AF700**/BV711	B-ly6	**(3)**/5/4	BD
**CD123**	**PerCP-Cy5.5**/BUV395	7G3	(3)5/4	BD
**CD14**	**BV650**	M5E2	5	Biolegend
**CD141**	APC/**BV510**	AD5-14H12/1A4	(3)/5	Miltenyi/BD
**CD16**	**FITC**/PE-Dazzle-594	3G8	5/(6)	BD/Biolegend
**CD19**	**FITC**/PERCPCy5.5/AF700	4G7/SJ25C1/H1B1	5/(5)/4	BD/BD/Biolegend
**CD1c**	**PE-Cy7**/PERCPCy5.5	L161	5/4	Biolegend
**CD2**	**PE**	RPA-2.10	5	BD
**CD20**	**FITC**/AF700	L27/2H7	5/3	BD/Biolegend
**CD3**	**FITC**/AF700	SK7(Leu4)	5/3	BD/Biolegend
**CD303**	**APC**/BV605	201A	5/4	Biolegend
**CD304**	**APC**/BV605	12C2/U21-1283	5/4	Biolegend/BD
**CD33**	**BV711**	WM53	5	Biolegend
**CD34**	**BV605**/APCCy7	581	5/(3)	Biolegend
CD4	PE	SK3 (Leu3a)	(5)	BD
CD45	AF700	HI30	(1)	Biolegend
**CD45RA**	**APCCy7**	HI100	5	Biolegend
**CD5**	**BUV730**	UCHT2	5	BD
**CD56**	**BV421**/APC	B159/NCAM16.2	5/(3)	BD
**CD7**	**FITC**	M-T701	5	BD
CD8	APC-Cy7	SK1	(3)	BD
**HLA-DR**	**BV785**/V500	L243/G46-6	5/(5)	Biolegend/BD
IFN-α	PE	LT27:295	10	MACS Milteyni Biotec
IKZF1	BV421	16B5C71	5	Biolegend
IL-10	APC	JES3-9D7	5	Biolegend
IL-12p40/p70	BV421	C8.6	5	BD
IL-1b	FITC	JK1B-1	5	Biolegend
IL-8	PE-Cy7	E8N1	5	Biolegend
TNF	APCCy7	Mab11	5	Biolegend

Supplementary table 2: Flow cytometry antibodies

Antibody, fluorochromes, clone, dilution and manufacturer of antibodies used in flow cytometry. Antigens and fluorochromes in bold text comprise the panel used for FlowSOM analysis. In the dilution column, plain text denotes the volume of antibody (μl) added to 50μl of buffer for staining cell suspensions; numbers in parentheses denote the volume of antibody (μl) added to 200μl whole blood (WB) in the Trucount™ analyses.

Supplementary table 3: Lenalidomide/Pomalidomide treatment patient details

Sampled	Age	Sex	Diagnosis	Lenalidomide Dose (mg od)	Dexamethasone dose (od x1/week)	Previous treatments
**On lenalidomide**	56	M	MM	15	20mg	6
	44	F	HD	15		4
	55	M	MM	15		5
**1 week off lenalidomide treatment**	67	M	MM	25	20mg	3
	43	M	MM	25	40 D1-4 + D15-18	4
	69	M	MM	25	10mg	3
	79	F	MM	15	10mg	0
	79	M	MM	15	2mg M,W,F	1
	83	F	MM	15	20mg D1-4	0
	84	M	MM	15	20mg D1-4	2
	74	F	MM	15	10mg	1
	56	M	MM	10		2
	61	F	MM	10		2
	54	M	MM	10		2
	48	F	MM	10	6mg	2
	63	F	MM	10	2mg D1-7	3
	72	F	MM	5		2
	79	M	MM	5		1
	83	M	MM	5		2
**1 week off pomalidomide**	54	M	MM	4	6mg weekly	4
	79	F	MM	4	20mg weekly	4

Supplementary table 3: Lenalidomide/Pomalidomide treatment patient details
Characteristics of patients on lenalidomide or pomalidomide treatment. For all patients, lenalidomide was taken daily for three out of four weeks, according to standard protocols. Sixteen patients were tested after seven days without lenalidomide (end of four week treatment cycle), three had taken lenalidomide in the previous twenty four hours ('On lenalidomide'). Dexamethasone was taken once a week for three out of four weeks, unless otherwise stated. All samples were taken a minimum of seven days after the last steroid dose. Pomalidomide treatment was taken daily for three out of four weeks with a single weekly dexamethasone dose. D, Day of four week cycle; M,W,F, Monday, Wednesday, Friday; MM, multiple myeloma; HD, Hodgkin's Disease.
